# Outcomes of Dental Implants in Routine Clinical Practice: A Retrospective Multicenter Study

**DOI:** 10.1155/ijod/9930477

**Published:** 2025-02-23

**Authors:** Funda Goker, Pooja Mali Rai, Daniele De Santis, Maurizio Colombo, Lorenzo Gornati, Emanuele Savoini, Sourav Panda, Massimo Del Fabbro

**Affiliations:** ^1^Department of Biomedical, Surgical and Dental Sciences, Università degli Studi di Milano, Milan, Italy; ^2^Department of Surgical, Dental and Maternal-Infant Sciences, University of Verona, Verona, Italy; ^3^Private Practice, Meda, Monza-Brianza, Italy; ^4^Private Practice, Cornaredo, Milano, Italy; ^5^Private Practice, Poirino, Torino, Italy; ^6^Department of Periodontics, Institute of Dental Sciences, Siksha ‘O'Anusandhan University, Bhubaneswar, India; ^7^Unit of Maxillo-Facial Surgery and Dentistry, Fondazione IRCCS Ca' Granda Ospedale Maggiore Policlinico, Milan, Italy

**Keywords:** dental implants, immediate implant, immediate loading, implant therapy, practice-based research

## Abstract

**Purpose:** This study evaluated the clinical and radiographic outcomes of subjects rehabilitated with dental implants placed in daily practice.

**Materials and Methods:** This retrospective multicentric case series study involved 339 patients (168 males, 171 females, mean age 54.0 ± 14.7 (standard deviation) years, range 28–81 years) treated in five private clinics, who received 651 implants between January 2019 and January 2023. The main outcomes were marginal bone loss (MBL) and implant survival rate. The effect of variables such as soft tissue status, crestal/subcrestal implant placement, immediate/delayed implantation, bone grafting/no graft, screwed, or cemented prosthesis were analyzed.

**Results:** The implant survival rate was 99.9%, with only one implant failure observed in a 71-year-old female with subcrestal placement. Implants immediately placed in post-extraction sites had significantly greater MBL compared to delayed placements (*p*=0.0002). Subcrestally positioned implants showed significantly less MBL than crestal implants (*p* < 0.0001), while grafted and non-grafted sites showed similar results, and cemented prostheses demonstrated lower MBL compared to screwed prostheses (*p* < 0.0001). The prevalence of peri-implant mucositis was only 3.6% on implant basis. No intra-operative complications nor adverse events in the follow-up period were reported.

**Conclusion:** Following current guidelines for implant therapy, consisting of proper diagnosis and personalised treatment plan and maintenance, and adhering to implant system manufacturer's recommendations, it is possible to achieve satisfactory clinical and radiographic outcomes in routine practice.

## 1. Introduction

Dental implants have evolved as a promising option for replacing missing teeth and have become routine practice in clinics over the past 40–50 years [1, 2]. Currently, it is proved that pure titanium implants can osseointegrate without adverse reactions in the surrounding living tissues [3, 4]. Implant treatment has continued to evolve through research and development over decades, with well-documented results about long-term survival and success [2].

Advancements in implant design and materials have led to extensive use of implants in various clinical scenarios such as immediate loading and placement in the most compromised sites. However, with the increasing use of dental implants in the clinics, new challenges like peri-implantitis and peri-implant bone loss have emerged. In every scenario, the stability of dental implants at the marginal level is one of the critical factors for successful long-term treatment outcomes [5]. Peri-implant diseases are defined as inflammatory lesions occurring in tissues surrounding dental implants, and clinical studies have demonstrated the prevalence of peri-implantitis ranged from 11% to 47% depending on marginal bone loss (MBL) thresholds [6]. A long-term with 20 years follow-up period and a large sample size (4206 patients and more than 12,500 implants) showed that sandblasted surface implants can have a cumulative survival rate of 93.3%, with most failures occurring within a year after placement due to failed osseointegration in the short term. In this same study, 2.6% of implants were found to have failed because of peri-implantitis [7]. Other clinical studies have also concluded that unaesthetic results such as gingival recession increase in middle- to long-term follow-ups [8]. Various systematic reviews have revealed that immediate implants may result in advanced gingival recession leading to aesthetic failure [9, 10].

The role of systemic diseases and periodontitis was also extensively investigated in literature as a cause of implant failures [11–13]. According to the literature, there is some evidence that patients treated for periodontitis can have a higher incidence of implant failures and complications [11]. However, there are also studies that mention similar outcomes and conclude that under strict maintenance programme, placing implants in patients with a history of periodontal disease is a safe treatment option with predictable results even in cases of short implants [12, 13]. The relationship between peri-implantitis and systemic disease is less known. In a recent literature review, it was concluded that there is no association between the risk of peri-implantitis and diabetes, cardiovascular disease, hypertension, or osteoporosis. However, a positive association was found for obesity [14]. The width of keratinised tissue was also shown to have an important effect on the increased prevalence of peri-implantitis, soft tissue inflammation and MBL [15–18]. Despite the continuous efforts to understand the reasons behind the bone loss around the implant, it is difficult to predict. According to recent literature, the preservation of crestal bone is one of the crucial factors influencing the success rate [19, 20]. On the other hand, a meta-analysis of randomised controlled studies reported that bone loss and high survival rates were not significantly different for crestal or subcrestal implant placements [20]. However, for soft tissue, health and success subcrestal placement seems to be preferred [19, 20].

In the literature, the survival rates of implants were tested in different clinical settings with different timing of placements, techniques, and surface materials [18–25]. Currently, immediate placement of implants has become a common choice for oral rehabilitation among dentists and patients globally. In a meta-analysis on comparison of the impact of immediate and delayed implant placement, the immediate placement was found to be slightly more associated with implant failures (with a success rate between 90%–95%) when compared with delayed (success rate of above 95%); however, these results were not statistically significant [21]. Traditionally, implant length of 10 mm is considered as an ideal length for implants, due to the idea that long implants have more surface area and lower crown–implant ratio which can be a favourable factor for success [23–25]. However, more recent studies have proved that successful results can also be obtained with short implants (6–8.5 mm), which can be as useful as standard long implants, in terms of survival rates, crestal bone loss levels, and prosthesis survival rates [23–25].

The majority of the studies in the literature on the success and survival rates of dental implants are conducted in university or hospital settings, with prospective studies, often controlled and randomised, performed by experienced and well-equipped teams, using up-to-date materials and techniques, and adopting strict selection criteria for reducing variability among patients included and follow-ups. Currently, the studies performed in routine dental clinics, reporting the actual results of the daily practice, are very limited in number. In the modern world, implant dentistry has become a routine part of dental clinical practice, and it is important to evaluate the outcomes of implant insertions in 'real life' other than centres such as university hospitals. This multicenter retrospective clinical case series study aimed to evaluate the outcomes of oral rehabilitation of edentulous patients who were treated with dental implants according to the daily routine protocols in five different dental clinics. The patients were followed for up to 68 months and primary evaluation based on the survival of dental implants and MBL around implants.

## 2. Materials and Methods

This retrospective research involved patients treated at five private clinics by five experienced clinicians. Institutional Review Board approval of the Fondazione IRCCS Ca' Granda Ospedale Maggiore Policlinico, Milan, Italy was obtained for retrospective studies on implant therapy, with number 420/01, RC2024. Declaration of Helsinki on medical protocol and ethics principles was followed in this work. The consent form with signatures was obtained from all patients for the treatment and use of data for scientific purposes. All the patients were treated according to the manufacturer instructions for implant placement. The surgical procedures of this study, as well as the patients' selection criteria, followed the national guidelines for implant rehabilitation, and were similar to those used in a previous study published by the same team of authors using the same implant brand (Dental Tech S.r.l., Misinto, MP, Italy) [26]. The implants were inserted between January 2019 and January 2023, and the patients are still at their routine follow-up. A brief description of the rehabilitation protocol is described below.

### 2.1. Patient Selection

Patients who received oral rehabilitation with dental implants with at least 1 year follow-up were included.

#### 2.1.1. Inclusion Criteria

• Adult patients with the ability to sign an informed consent form;
• Patients who received at least one dental implant;
• Patients with a minimum 1 year of follow-up after implant insertion who had bone loss evaluation after 1 year of implant placement;
• Healthy patients according to the criteria of the American Society of Anesthesiologists (ASA-1 or ASA-2), that declared to have no contraindication for implant surgery.

Diabetes under control, osteoporosis, bruxism or smoking habits were not considered as a contraindication.

#### 2.1.2. Exclusion Criteria

• Oncological patients, patients under chemotherapy/radiotherapy;
• Pregnant subjects;
• Patients under anti-resorptive medications (e.g., bisphosphonate, denosumab);
• Patients suffering from acute periodontitis/gingivitis and/or active infection in head/neck or mouth which can have a negative effect on implant survival.

This study evaluated the results of titanium grade 5 (Ti_6_Al_4_V) implants with sandblasted and acid-etched surface (BWS, Blasted Wrinkled Surface), produced by Dental Tech S.r.l. (Misinto, MP, Italy). Due to a specific manufacturing process (sandblasting with pure aluminium oxide followed by etching with hydrofluoric and nitric acid), these implants are characterised by an extremely clean surface, with uniform and homogeneous roughness, potentially favouring fibrin and cell adhesion. The codes and brief explanations of the implant used in this study are listed as follows: IMPLASSIC FT3 (cylindric implant with conic apex 3.25–4.75 mm diameter, 8–16 mm long), IMPLASSIC FT2 (submerged, cylindric short implant (6–7 mm) with tapered apex), IMPLASSIC® TR2 (transmucosal, cylindric implant; internal design: cone with anti-rotational octagon), IMPLASSIC TRW2 (transmucosal, cylinder implants with wide prosthetic platform *Ø* 6.5 mm), IMPLASSIC® TR3 (transmucosal implant with atraumatic apex; internal design: cone with anti-rotational octagon) and IMPLOGIC® AT (submerged, conical implants, active thread in cases immediate implantation at post-extraction sites, 3.25–4.75 mm diameter, 8–13 mm long).

All patients had surgical interventions according to the surgical recommendations of the manufacturer (Dental Tech S.r.l., Misinto, Italy). Bone grafts and absorbable haemostatic gelatin sponge were placed in cases of need for post-extraction alveolar ridge preservation. The list of grafts and dressings used in this study is Spongostan™ Dental (Ethicon, Sweden), Exaflex resorbable membrane (GC International AG, Italy) and MimetikOss™, Creos™ Syntogain Joins Creos™ Syntoprotect, Creos Xenoprotect, Creos Xenogain and Creos Mucogain (Nobel Biocare, Switzerland).

### 2.2. Surgical Protocol

The first step was the general and oral health anamnesis information collection. The patients were clinically examined, diagnostic radiographs were evaluated and the patients were operated on following general guidelines for implant therapy and rehabilitation, as also described previously [26]. For prophylaxis, antibiotics were prescribed according to current guidelines. On the day of surgery, a full thickness incision was utilised in the region of interest using #15c surgical scalpel. Following the primary incisions, flaps were elevated, implant sites were prepared and implants were inserted according to the manufacturer's instructions. In cases of need, appropriate grafts (xenograft/allograft or autologous bone) and collagen membranes were used. The flaps were repositioned and sutured with single interrupted sutures using non-resorbable silk sutures. Follow-up visits were planned as at 1, 3, 6, 12 months and yearly thereafter.

### 2.3. Evaluation of Data

Primary outcomes were dental implant survival after at least 1 year of function, and marginal bone level (MBL) change around implants. The latter was calculated utilising periapical radiographs [26]. The baseline value corresponded to the measurements taken at prosthesis delivery. These were compared with those taken at subsequent follow-ups, at least 1 year after implant insertion and yearly thereafter. The difference between measurements taken at follow-up and at baseline was considered as the MBL change at a given time. The measurements were taken at the mesial and distal aspect which was calculated as an average single value for each implant. In case of patients with multiple implants, a single per patient value was also obtained. The software ImageJ (National Institutes of Health, USA) was used for measurements. The implant length and diameter served for calibration. MBL changes were measured at each centre by an evaluator with expertise in radiographs assessment. Measurements were then transmitted from each centre to the statistician. The results were summarised as mean bone level changes (in mm) ± standard deviation (SD). In the clinical follow-ups, patients were re-evaluated, and each data were noted on patient record with dates [26]. Peri-implant soft tissue status was noted as healthy or mucositis/peri-implantitis. All the clinics of this study adopted the criteria established in the 2017 World Workshop on the Classification of Periodontal and Peri-Implant Diseases and Conditions for defining periodontal and peri-implant cases [27].

### 2.4. Statistical Analysis

Descriptive statistics of the collected data was performed. The quantitative variables were summarised by using mean values and standard deviation when data were normally distributed. The normality of distributions was evaluated using the D'Agostino and Pearson omnibus normality test. Non-gaussian distributions were summarised by using the median and 95% confidence intervals. Dichotomous variables were expressed using absolute and relative frequencies (percentages). The following confounding variables were evaluated as potentially affecting MBL, failures and complications: soft tissue status, crestal/subcrestal implant placement, immediate/delayed implantation, bone grafting/no graft and screwed or cemented prosthesis. The effect of each variable on MBL was evaluated using unpaired Student's *t*-test or Mann–Whitney test, according to the type of distribution. The comparisons regarding implant failure (loss) or complications were undertaken by using Pearson's chi square or Fisher's exact test, as appropriate (the Fisher's test was used when the absolute frequency of an event was less than 5). The reason for extraction (caries, periodontitis, fracture) was also considered, and MBL was evaluated and compared in these different situations, respect to edentulous sites, using Kruskal–Wallis test followed by Dunn's Multiple Comparison Test. The implant was considered as the unit of analysis. The significance level was set at *p*=0.05. Statistical analysis was undertaken with GraphPad Prism 5.03 (GraphPad Software, Inc., La Jolla, CA, USA).

## 3. Results

This research included 339 patients (168 male and 171 female) who had 651 implants inserted at five different dental clinics. Mean age of the study group was 54.0 ± 14.7 years (age range: 28–81 years, median age: 60 years). Regarding the medical status of the study population three patients were suffering from osteoporosis (they were taking bisphosphonates as medication), one patient had lichen planus, one had type 2 diabetes (taking metformin), 14 had hypercholesterolaemia and 49 patients had hypertension (taking anti-hypertension drugs), while 271 patients were reported as medically healthy. There were 49 smokers (range 5–40 cigarettes per/day: 3 patients declared to smoke 40 cig/day, 5 patients 30/day, 4 patients 25/day, 11 patients 20/day, 9 patients 15/day, 9 patients 10/day, 8 patients 5/day). There was one alcoholic subject with drinking habit of 12 glasses/day.

The mean follow-up period from the date of the implant surgery was 45.95 ± 12.01 months (minimum 20.04 and maximum 68.27 months). The mean follow-up period from the date of loading with prosthesis was 44.42 ± 11.61 months (minimum 3.05 and maximum 66.69 months). The mean healing period from the date of the implant surgery and the date of loading was 3.38 ± 2.48 months (minimum 0 for immediate loading cases and maximum 22.28 months).

**Figure 1:**
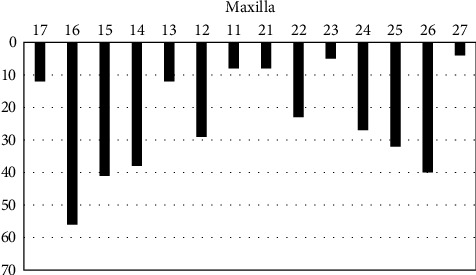
Number of implants placed in the different sites (17–27) for maxilla.

**Figure 2:**
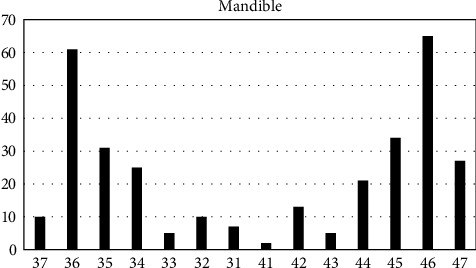
Number of implants placed in the different sites (37–47) for mandible.

The implant distributions per site of implant insertion are listed in Figure 1 for maxilla and Figure 2 for mandible. The dimensions of implants that were inserted are listed in Table 1. Tables 2 and 3 shows bone loss in mm and comparisons among variables. The implant numbers for the reason of extraction, immediate implantation or delayed protocol (at least 6 months after extraction), additional grafting or not, periodontal health situation after implant insertion (mucositis versus healthy peri-implantitis tissues, crestal or subcrestal implant insertion, can also be found in detail in Tables 2 and 3.

Additional detailed data which were not included in the statistical evaluation: *Grafts:* 58 sites had xenografts placed (either equine, bovine or porcine origine), and one received alloplastic graft. One case had an additional autogenous graft for bone augmentation. Twenty-three patients had additional collagen membranes together with grafts, nine had haemostatic medications to control excessive bleeding and two patients received connective soft tissue grafts. *Antagonist dentition:* for 217 implants the antagonist was natural teeth, ceramic prosthesis: 77, full arch prosthesis: 38, edentulous: 3, monolithic disilicate: 32, Resin prosthesis: 45, Toronto: 218, veneer resin: 21. *Prosthetic material:* ceramic 282, Resin 113, vetro-ceramic (disilicate) 256. *Implant failure:* Only one subcrestally placed implant (Implassic FT3, size 3.75 × 8 mm, position 16) was lost after 1 year of function in a 71-year-old female patient with periodontal problems who received bone graft (Creos) and a resin prosthesis. The overall survival rate of implants was 99.9%. No statistical comparison was undertaken for implant survival because of the very small incidence of implant failures.

## 4. Discussion

The evolution and optimisation of implant protocols based on clinical experience and research progress in terms of materials, surfaces, production standards, and quality control have helped drastically to reduce the complications and adverse events in implant dentistry [28]. Currently, a considerable part of the implantology research focuses on risk assessment and prevention, especially through careful compliance with guidelines and procedures [29]. There is plenty of research in the literature about factors affecting MBL after implant insertion [30–33]. Albrektsson et al. [30] reported that implants with lower values of insertion torque and primary implant stability seem to be more prone to failure, particularly when using immediate or early loading protocols, compared to implants placed with higher primary stability. This can be explained by the different patterns of bone resorption that can occur in the first year after implantation, possibly due to bone remodelling, which is an adaptive bone response to both surgical trauma and a change in the mechanical forces to which bone tissue is exposed, once occlusal loading is applied [31]. A randomised controlled trial on outcomes of immediately loaded implants after 1-year follow-up found mean MBLs of 0.01 mm for immediately placed and 0.06 mm for delayed implants, being the difference statistically significant [32]. A study on the primary and secondary stability of implants showed lower MBL values in the delayed implant placement compared to immediate placement group [33]. After the remodelling phase, the MBL often stabilises for about 0.2 mm per year, and the overall MBL is generally lower for delayed implant insertions when compared to immediately placed implants [34]. Another study conducted by Karni et al. [35] found relevant differences in peri-implant probing depth and MBL in cases with immediate implantation (significantly higher values for immediate versus delayed placement). Additionally, it was stated that during the first year, the immediately placed implants have a tendency for faster bone remodelling [34, 35]. Nevertheless, several other factors such as deep implant insertion with respect to ridge level, thin peri-implant mucosa and short abutments also seem to influence negatively the MBL [36]. The results of this work were consistent with the literature, as the implants immediately inserted after tooth extraction showed significantly more bone loss at 1 year (even though the size of the difference was small, about 0.2 mm on average) than delayed protocol (implants placed after at least 6 months) [30–33].

A history of periodontal diseases can increase the risks of peri-implant mucositis and peri-implantitis, and implant failure [37, 38]. In this study, the incidence of mucositis was relatively low (3.6% at peri-implant level). However, subjects with healthy peri-implant tissues showed a slightly greater bone loss than patients with peri-implant mucositis (*p*=0.045). However, this finding should be considered very cautiously because of the great difference in the sample size (23 cases of peri-implant mucositis versus 616 implants with healthy surrounding periodontal tissues).

According to the literature, preservation of crestal bone is a crucial factor for the success of the implants [19, 39], and there are several reports comparing subcrestal and crestal implant insertions; however, the results are conflicting [19, 20, 40–44]. A recent meta-analysis showed a significantly greater MBL for crestal implants compared to subcrestal ones [20]. On the other hand, some studies showed higher bone loss for sub-crestal group [42, 43]. Another systematic review and meta-analysis assessed overall clinical outcomes and found clinically acceptable peri-implant tissue parameters for both approaches (crestal versus subcrestal placements) [44]. In this study, subcrestal insertions had less MBL than crestal ones, which was in accordance with the result of the above-mentioned meta-analysis [20].

According to the results of this research, bone grafting had lower MBL values compared to sites without any grafts (*p*=0.18). A meta-analysis investigated the hypothesis that there is an effect of different bone graft substitutes on implant stability when used in patients undergoing immediate implant surgery and concluded that the use of grafts and immediate implant placement resulted with an increased bone gain (which is in agreement with findings of this research) [45]. According to the reports in literature, autogenous grafts such as iliac bone grafts mixed with demineralised dentin matrix provide improved implant stability and satisfactory functional results compared to commercially available bone grafts [46, 47]. There are also a variety of reports about different types of grafts such as mixtures of xenografts (such as deproteinized bovine bone) and hydroxyapatite nanoparticles showing promising results for implant stability and bone remodelling [48, 49]. An animal study by Araújo et al. on Beagle dogs with 6 months follow-up showed that the group without bone graft had a vertical buccal bone loss of 1.3 mm, while the test group with bone graft showed no bone loss, suggesting that grafting can help to preserve vertical bone levels [50]. In a meta-analysis, authors have demonstrated that immediate implant placement combined with bone grafting can significantly contribute to ridge preservation, which aligns with the findings of this study [51]. Furthermore, there are several reports which highlight the positive outcomes of grafting during immediate implant placement for minimising horizontal buccal bone resorption and enhancing the aesthetics in the peri-implant soft tissues [52].

The interest of clinicians in immediate loading has been witnessed in the literature by a steadily growing number of publications in the past years [53–55]. Recent systematic reviews concluded that under well-defined circumstances, immediate loading seems to have long-term predictability and success rate [53–55]. Another popular topic in implantology is immediate implant placement, which brings certain advantages like reduced treatment time, less surgical interventions, and better preservation of hard and soft tissues [56–58]. However, it also brings certain disadvantages such as higher risks of implant failure with respect to delayed placement, especially in the first year following surgery [56]. This is mostly due to the lack of stability in the early phase at the extraction site. On the other hand, delayed implants can show some advantages such as higher primary stability and lower risk of infection due to implant placement in completely healed tissues [58]. These results are in accordance with the results of this study, since delayed implant placement showed less bone loss. However, delayed implantations as highlighted in the literature have disadvantages like the need for additional surgeries and aesthetic challenges due to changes in tissue contour during healing [56, 58]. According to a systematic review that assessed the tissue changes, immediate implants had significant advantages like better preservation of alveolar bone and reduced gingival recession, which adds aesthetic benefits, especially in the anterior region [51].

Regarding the prosthetic connection, the choice of screwed or cemented prostheses largely depends on clinician's choice for the individual case [59, 60]. A meta-analysis concluded that the cement-retained restorations showed lesser bone loss when compared to screwed [59], which is also in accordance with this study. Screw-retained restorations offer certain advantages like retrievability, passive fit and ease of repair, while one of the major disadvantages of them is risk of peri-implantitis due to residual cement [60]. Hamed et al. [22] compared cemented and screwed implants and concluded that screw retained implant-supported reconstructions were shown to have fewer technical and biological complications such as peri-implantitis.

According to a recent meta-analysis, a history of periodontitis significantly impacts the implant survival rate, peri-implantitis and mean bone loss [61]. However, the severity of periodontitis does not seem to influence the survival rate. In literature, a high survival rate and a comparable bone loss is reported both for crestal and subcrestal implants' placement regardless of the history of periodontal disease [19, 36, 43, 44]. However, in cases, when the patient has thin mucosal tissue, a subcrestal implant placement should be preferred, to prevent further risks of peri-implant pathologies [61]. In this study, just one subcrestally placed implant was lost within 1 year of function in a patient who had a history of periodontal problems and underwent bone grafting. The overall implant survival rate was 99.9%. No statistical analysis was done to evaluate the weight of factors affecting the survival of implants because of the very small number of implant losses.

One of the limitations of this study is the fact that data were obtained from patients treated in the daily practice in five different dental clinics. No specific procedures were imposed, so that the clinics were free to use their individual protocols for diagnosis, treatment and follow-up assessment. In spite of the same data collection form being sent to the referent at each clinic, and explanations were given on how to fill it, the files returned with some information missing and non-uniform terminology and format used to fill the cells, which required an additional effort for standardising and preparing the data for the analysis. However, this study represented a situation truly close to real life, which is different from the situation encountered in standard prospective clinical trials, randomised or not. The latter are usually performed at university clinics, by experienced clinical research teams, using rigorous methodology and up-to-date procedures and materials. All steps to be followed during the research are specified in predetermined protocols, and all outcomes are collected in a standard way, carefully and precisely, using specific tools and procedures. In these studies, the inclusion and exclusion criteria are clearly specified before starting and are often very selective to minimise sample variability and met the aim of the study, testing the working hypothesis with the highest possible precision. Given the features of clinical trials, such studies usually can determine the effect of a given treatment in the ideal situation. The latter, however, can hardly be compared to the daily practice, and the results are expected to be different as well. It is difficult to say if in daily clinical practice the outcomes should be better or worse, but certainly the variability is higher than in clinical trials, mainly due to the absence of specific restrictions in the selection criteria. However, if we look at the external validity (which means how the findings of a given study may be transferred to real life), the results of this study can be considered as strong, since these outcomes were obtained from routine clinics, mirroring the reality in everyday practice. Other studies evaluated the outcomes of dental implant treatment in general practice setting, reporting that implant success rates can be lower than those achieved in well-controlled clinical trials performed in university clinics [62, 63]. What was common in the five dental clinics of this study is that clinicians strictly followed the manufacturer's instruction for surgical and prosthetic procedures and current clinical guidelines for implant treatment, achieving extremely satisfactory results in terms of implant survival and MBL. Of course, such short-medium-term results must be confirmed with a longer follow-up. Another limitation, also due to the retrospective nature of this study, is the absence of patient-reported outcome measures and specific evaluation of the aesthetic aspect using standardised tools, which would have strengthened the evaluation of the treatment.

## 5. Conclusion

This study can be interesting to the readers since it represents the real-world situation at general dental practices. According to the authors of this work, following current guidelines, proper diagnosis and patient-specific treatment plan can help to achieve successful oral rehabilitation outcomes for dental implants in ordinary clinics. However, further studies with longer follow-up periods are needed to confirm the present results.

## Figures and Tables

**Table 1:** Number and dimensions of implants that were inserted (in mm).

Implant dimensions	Length 6	Length 8	Length 10	Length 11.5	Length 13	Length 16
Width 3.25	0	5	2	12	7	2
Width 3.75	0	18	103	217	64	7
Width 4.25	9	47	77	42	17	3
Width 4.75	0	12	6	1	0	0

**Table 2:** Bone loss in mm and comparison among variables with *t* test. For some implants the information was missing.

Parameter	Soft tissue status		Implant positioning		Implant insertion		Grafting		Prosthesis connection	
	Mucositis	Healthy	Crestal	Subcrestal	Immediate	Delayed	Yes	No	Screwed	Cemented
Number of implants	23	616	120	317	169	470	59	580	334	297

Total	639		437		639		639		631	

Median	0	0.1	0.5	0	0.3	0	0	0,1	0,3	0
Mean	0.1261	0.3221	0.5483	0.1959	0.4302	0.2736	0.3237	0.3141	0.4401	0.1694
SD	0.1864	0.4357	0.3149	0.4797	0.5278	0.3822	0.5975	0.4107	0.4956	0.2416
Lower 95% CI of mean	0.04548	0.2876	0.4914	0.1429	0.35	0.239	0.168	0.2806	0.3868	0.1418
Upper 95% CI of mean	0.2067	0.3566	0.6053	0.2489	0.5103	0.3083	0.4794	0.3476	0.4935	0.1969

Mann–Whitney test (*p*-value)	0.0449		<0.0001		0.0002		0.1758		<0.0001	

Abbreviation: SD, standard deviation.

**Table 3:** Analysis of marginal bone loss according to the reason for extraction or edentulism status.

Parameter	Caries	Fracture	Edentulous	Periodontitis
Number of values	240	46	281	71

Sum	638			

Median	0	0.6	0.2	0.2
Mean	0.1758	0.6609	0.3498	0.4239
Std. deviation	0.2654	0.4394	0.4315	0.6475
Lower 95% CI of mean	0.1421	0.5304	0.2991	0.2707
Upper 95% CI of mean	0.2096	0.7914	0.4005	0.5772

Dunn's multiple comparison test (*p*-value)		<0.0001		

**Comparison**		**Difference in rank sum**	**Significance (** **p** < 0.05**)**	**Summary**

Caries vs fracture		−208.5	Yes	*⁣* ^*∗∗∗*^
Caries vs missing		−75.44	Yes	*⁣* ^*∗∗∗*^
Caries vs periodontitis		−64.64	Yes	*⁣* ^*∗*^
Fracture vs edentulous		133.1	Yes	*⁣* ^*∗∗∗*^
Fracture vs periodontitis		143.9	Yes	*⁣* ^*∗∗∗*^
Edentulous vs periodontitis		10.8	No	ns

Abbreviations: CI, confidence interval; ns, non-significant.

*⁣* ^*∗*^: significant; *⁣*^*∗∗∗*^: highly significant.

## Data Availability

The data that support the findings of this study are available from the corresponding author upon reasonable request.
